# Assessing the impact of using a patient counselling prompt—the TARGET antibiotic checklist in England’s community pharmacies

**DOI:** 10.1093/jacamr/dlaf018

**Published:** 2025-02-19

**Authors:** Sejal Parekh, Lingqian Xu, Catherine V Hayes, Kieran Hand, Diane Ashiru-Oredope, Donna M Lecky

**Affiliations:** Primary Care Strategy and NHS Contracts Group, Primary Care, Community Services and Personalised Care Directorate, NHS England, London SE1 8UG, UK; Primary Care Strategy and NHS Contracts Group, Primary Care, Community Services and Personalised Care Directorate, NHS England, London SE1 8UG, UK; HCAI, Fungal, AMR, AMU & Sepsis Division, UK Health Security Agency, London SW1P 3HX, UK; AMR Programme, Medical Directorate, NHS England, London SE1 8UG, UK; HCAI, Fungal, AMR, AMU & Sepsis Division, UK Health Security Agency, London SW1P 3HX, UK; HCAI, Fungal, AMR, AMU & Sepsis Division, UK Health Security Agency, London SW1P 3HX, UK

## Abstract

**Background:**

An estimated 1.27 million deaths globally were caused by antibiotic-resistant infections in 2019. Outcome 2 of the UK national action plan to combat antimicrobial resistance is improved public engagement and education with a specific and measurable target.

**Objectives:**

To evaluate and compare 2 years of the use of the TARGET antibiotic checklist in England’s community pharmacies via the Pharmacy Quality Scheme (PQS).

**Methods:**

The use of the TARGET antibiotic checklist was incentivized in the PQS for 2021–22 and 2023–24 for patients presenting with antibiotic prescription in community pharmacy during a 4 week period each year.

**Results:**

A total of 406 333 patients were counselled using the TARGET antibiotic checklist, with 10 081 community pharmacies participating in either year and 6209 community pharmacies participating in both years. The most common indications for both years were chest and urinary tract infections with amoxicillin and nitrofurantoin, respectively, being the most frequently prescribed antibiotics for both PQS years examined. A total of 27 898 influenza vaccinations were delivered by community pharmacies prompted by discussions whilst using the antibiotic checklist. In addition, 140 473 patient information leaflets were provided to patients to improve knowledge about their condition and treatment and to support future self-care.

**Discussion:**

The investment in training and resources for community pharmacies through the PQS has provided opportunities for strengthening antimicrobial stewardship by equipping them with the tools to improve patient knowledge of antibiotic use, symptom resolution and antimicrobial resistance using the TARGET antibiotic checklist, as well as other resources from the TARGET Antibiotics Toolkit.

## Introduction

Drug resistance arises when microbes develop the ability to resist the antimicrobials designed to kill them, undermining treatment that can be offered to patients.^1^ The UK’s 20 year vision for antimicrobial resistance (AMR) is a world where AMR is effectively contained, controlled and mitigated against.^2^ Much progress has been made from the UK’s 2013–18 resistance strategy.^3^ Despite this, an estimated 4.71 million deaths globally were caused by resistant infections in 2021.^4^ AMR continues to increase in the UK, where an estimated 7580 deaths were attributable to resistant infections and 35 200 deaths were associated with resistant infections in 2019.^1,3^

In 2022, the largest proportion of UK antibiotic prescribing was generated through general practice (80.2%), and penicillins such as amoxicillin were the most frequently consumed antibiotics in England.^5^ The UK developed the national action plan (NAP) ‘Confronting antimicrobial resistance 2024 to 2029’, which builds on the achievements and lessons from the first 5 year NAP for AMR.^1^ This action plan outlines nine strategic outcomes organized under four themes for action that will be taken across all sectors including human health, animal health, agriculture and the environment.^1^ The first theme from the NAP focuses on reducing the need for and unintentional exposure to antimicrobials through public engagement and education on the risk of exposure to antimicrobials.^1^

**Figure 1.**
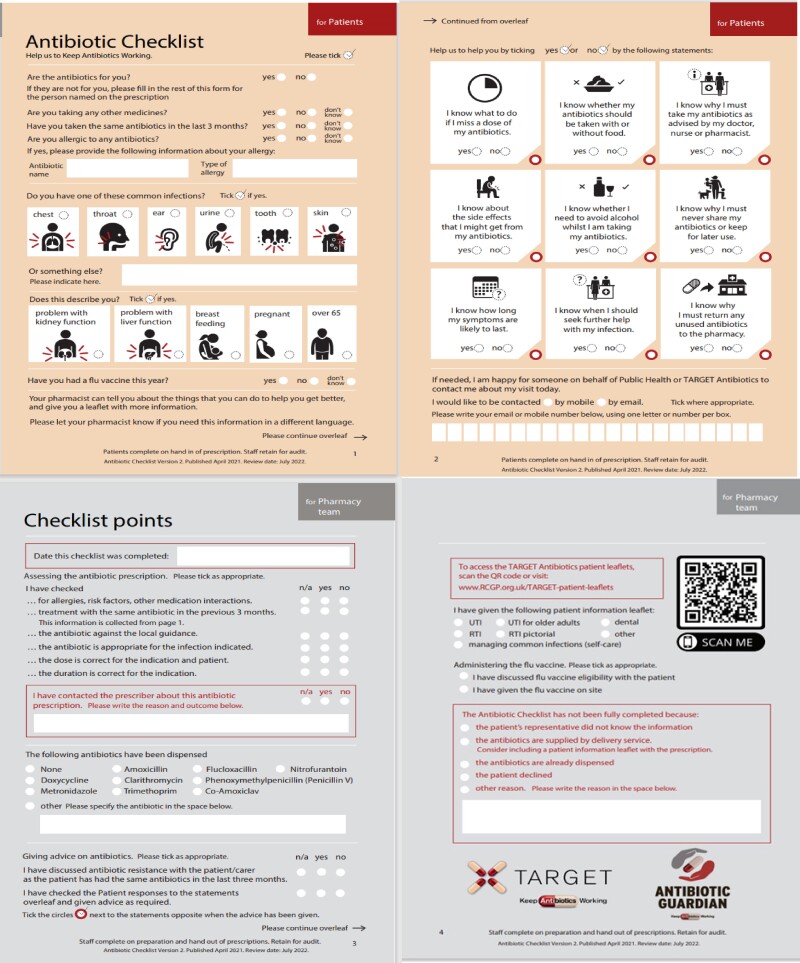
TARGET antibiotic checklist used in community pharmacy.

The UK Health Security Agency (UKHSA) leads the TARGET (Treat Antibiotics Responsibly, Guidance, Education and Tools) antibiotics toolkit, which consists of evidenced-based resources for primary healthcare professionals to enable appropriate antimicrobial prescribing and stewardship (AMS) and shared decision-making with patients.^6^ The TARGET antibiotic checklist (Figure 1) was designed to be used by community pharmacy teams as a prompt to check the appropriateness of the antibiotic choice and dose for the patient’s infection, and to facilitate individually tailored advice to patients/carers about their treatment following receipt of an antibiotic prescription.^7,8^

Community pharmacists are the third largest group of healthcare professionals globally; they are responsible for the safe and effective use of all medicines.^9^ There were 80 000 registered pharmacists in the UK in 2023.^10^ In England, the role of community pharmacists has been expanded to provide some health services that historically would have been delivered by GPs.^11^ In 2021–22, the use of the TARGET antibiotic checklist was incentivized as part of the Pharmacy Quality Scheme (PQS)—a national incentive scheme administered by NHS England.^8^ In the subsequent year, 2022–23, the use of the TARGET ‘treating your infection’ leaflets for both urinary and respiratory tract infection was incentivized as part of the PQS.^12^ In 2023–24, the PQS re-incentivized the use of all three TARGET tools (TARGET antibiotic checklist and both ‘treating your infection’ leaflets for urinary and respiratory tract infections). This study evaluates and compares the use of the TARGET antibiotic checklist during the PQS 2021–22 and the PQS 2023–24.

## Materials and methods

### Study design

This was a service evaluation and comparison of the implementation of the TARGET antibiotic checklist resource in community pharmacies assessed via the national PQS incentive scheme. The materials and methods have been previously described in ‘The use of the TARGET antibiotic checklist to Support Antimicrobial Stewardship in England’s Community Pharmacies’.^8^

### Setting and participants

In March 2023, there were 11 043 registered community pharmacies in England.^13^ The PQS is voluntary, and all NHS community pharmacy contractors in England can participate.

### Data collection

For the 2023–24 PQS, there were amendments to the data collection tool to make all questions compulsory and prevent blank responses/bypassing them to the next screen. The online snap survey portal (Snap 11 Professional) digitalized the questions from the TARGET antibiotic checklist, as well as automatically capturing demographic information such as the pharmacy’s Organisation Data Service (ODS) code and address. A single form submission was required per prescription. For the 2023–24 PQS, pharmacy teams collected data during 4 weeks between 1 June 2023 and 31 March 2024. Each participating pharmacy could collect data independently during that time period. The data were collected using the NHS Business Services Authority (NHSBSA) snap survey, which was accessed via the Manage Your Service (MYS) software application before making the PQS declaration of participation.

### Data analysis

For the 2023–24 PQS, data were extracted from the NHSBSA MYS system following submission by community pharmacy contractors. As the data collection tool was amended for 2023–24, responses such as ‘no response’ were removed from the 2021–22 data to allow a fair comparison for parts of the analysis. The data were analysed descriptively using R statistical software version 4.2.1 (R Foundation for Statistical Computing). The chi-squared test of independence, which compares equality of proportions, was used to test for significant differences between 2021–22 and 2023–24. The results were reported as frequencies (and percentages) and *P* values for differences between the proportions for the two timepoints. A *P* value of less than 0.05 was considered statistically significant.

### Ethics

NHS ethical approval was not required as this study was a service evaluation. NHSBSA, who designed the data collection tool, provided contractors with information for the purpose of collecting data and that data would be collected primarily as a method of commissioner assurance to be able to confirm completion of PQS. No patient-identifiable data were submitted. All data were managed in accordance with the Data Protection Act 2018 and the General Data Protection Regulations (GDPR). All community pharmacies consented to participate through the online portal.

## Results

A total of 406 333 patients were reviewed using the TARGET antibiotic checklist across both years of the PQS, with 10 081 (8363 for 2021/22 and 7927 for 2023/24) community pharmacies participating in either PQS scheme and 6209 community pharmacies participating in both PQS schemes. In 2021–22, 213 105 TARGET antibiotic checklists were submitted and 197 475 checklists were submitted in 2023–24.

### Patient-reported indication and antibiotic prescribed

Most patients were collecting antibiotics for themselves [78% (163 446) in 2021–22 and 79% (155 697) in 2023–24], of which approximately half were taking other medication simultaneously [51% (107 360) in 2021–22 and 48% (93 949) in 2023–24]. Twelve percent (25 574) of patients reported an allergy to an antibiotic in 2021–22 and 6% (12 083) in 2023–24 (Table 1). The most common indications presenting for antibiotics for both years were chest and urine infections (Table 2). With the exception of dental infections, which fell from 15% (30 764) to 12.1% (24 440), of all requests, other infections seem consistent in both PQS years (Table 2). The reduction in dental infections aligns with a reduction in metronidazole prescriptions from 6.5% (13 720) in 2021–22 to 5.2% (11 804) in 2023–24, whilst the slight increase in throat infections aligns with an increase in phenoxymethylpenicillin (Penicillin V) prescribing from 6.5% (13 535) to 8.2% (16 260), typically used to treat bacterial sore throats. The most frequently prescribed antibiotics for both PQS years were amoxicillin and nitrofurantoin.

**Table 1.** Findings from patient section of the TARGET antibiotic checklist

	Were the antibiotics for the individual collecting? *n* (%)		Was the patient taking any other medicines? *n* (%)		Was the patient allergic to any antibiotics? *n* (%)	
	2021–22	2023–24	2021–22	2023–24	2021–22	2023–24
Yes	163 446 (78)	155 697 (79)	107 360 (51)	93 949 (48)	25 574 (12)	12 083 (6)
No	42 123 (20)	41 778 (21)	90 764 (43)	94 777 (48)	165 383 (79)	171 521 (87)
Do not know/missing	1954 (0.9)	N/A	8307 (4.0)	8749 (4)	14 867 (7.1)	13 871 (7)
No response	1335 (0.6)	N/A	2427 (1.2)	N/A	3034 (1.5)	0
Total	208 858 (100)	197 475 (100)	208 858 (100)	197 475 (100)	208 858 (100)	197 475 (100)

**Table 2.** The frequency of infection type and antibiotic prescribed reported by patients collecting antibiotic prescriptions

	Number of checklists, *n* (%)	
	2021–22	2023–24
Indication		
Chest	62 544 (30.5)	67 616 (33.5)
Urine	43 093 (21.0)	43 644 (21.6)
Tooth	30 764 (15.0)	24 440 (12.1)
Skin	23 091 (14.9)	21 384 (10.6)
Throat	22 513 (11.0)	23 784 (11.8)
Ear	13 367 (6.5)	12 509 (6.2)
Other	10 884 (5.3)	8726 (4.3)
Total^a^	205 256 (100)	202 103 (100)
Antibiotic prescribed		
Amoxicillin	73 921 (35.2)	66 137 (33.2)
Nitrofurantoin	30 459 (14.5)	31 820 (15.6)
Flucloxacillin	24 093 (11.5)	21 507 (10.8)
Doxycycline	19 420 (9.3)	21 555 (10.9)
Metronidazole	13 720 (6.5)	11 804 (5.2)
Phenoxymethylpenicillin	13 535 (6.5)	16 260 (8.2)
Clarithromycin	11 473 (5.5)	10 225 (5.1)
Other	9151 (4.4)	7119 (3.5)
Trimethoprim	7572 (3.6)	7414 (3.7)
Co-amoxiclav	6493 (3.1)	5560 (2.8)
Total^a^	209 837 (100)	199 401 (100)

^a^Some checklists had two indications selected, whilst some patients had more than one antibiotic prescribed simultaneously. Blank data fields (12 164) from the 2021–22 dataset were removed from the denominator when calculating percentages.

### Patient-reported knowledge on their infection and antibiotic use

The checklist presented patients with statements about their antibiotic course and infection treatment to assess their knowledge. Perceived knowledge appears to be high (at least 75% of respondents selected the ‘yes’ option) for all statements and these were broadly similar across both years with a marginal increase for some statements in 2023–24 (Table 3).

**Table 3.** Patient self-reported knowledge of specific information relevant to infection and safe and effective antibiotic treatment (knowledge of antibiotic use, knowledge about symptom resolution, antimicrobial stewardship)

	Patient response 2021–22, number of checklists, *n* (%)				Patient response 2023–24, number of checklists, *n* (%)				
Statement	Yes, I understand	No, I do not understand	No response	Total	Yes, I understand	No, I do not understand	No response	Total	*P* value for ‘Yes, I understand’
I know what to do if I miss a dose of my antibiotics	166 519 (79.8)	25 953 (12.4)	16 143 (7.7)	208 615 (100.0)	156 628 (79.3)	28 646 (14.5)	12 201 (6.2)	197 475 (100)	<0.001
I know whether my antibiotics should be taken with or without food	159 296 (76.4)	32 247 (15.5)	17 065 (8.2)	208 608 (100.0)	152 115 (77.0)	33 196 (16.8)	12 164 (6.2)	197 475 (100)	<0.001
I know why I must take my antibiotics as advised my doctor, nurse or pharmacist	184 511 (88.5)	8809 (4.2)	15 270 (7.3)	208 590 (100.0)	174 668 (88.5)	10 742 (5.4)	12 065 (6.1)	197 475 (100)	0.9593
I know whether I need to avoid alcohol whilst I am taking my antibiotics	162 809 (78.1)	27 135 (13.0)	18 579 (8.9)	208 523^a^ (100.0)	154 203 (78.1)	28 829 (14.6)	14 443 (7.3)	197 475 (100)	<0.941
I know about the side effects that I might get from my antibiotics	155 845 (74.8)	34 560 (16.6)	18 079 (8.7)	208 484^a^ (100.0)	149 275 (75.5)	35 272 (18.1)	12 928 (6.5)	197 475 (100)	<0.001
I know how long my symptoms are likely to last	157 845 (75.7)	32 247 (15.5)	18 425 (8.8)	208 517^a^ (100.0)	152 023 (77.0)	31 690 (16.0)	13 762 (7.0)	197 475 (100)	<0.001
I know when I should seek further help with my infection	176 540 (84.6)	14 656 (7.0)	17 406 (8.3)	208 602^a^ (100.0)	168 274 (85.2)	16 308 (8.3)	12 893 (6.5)	197 475 (100)	<0.001
I know why I must never share my antibiotics or keep for later use	179 888 (86.3)	12 030 (5.8)	16 644 (8.0)	208 562^a^ (100.0)	169 588 (85.9)	14 671 (7.4)	13 216 (6.7)	197 475 (100)	<0.001
I know why I must return any unused antibiotics to the pharmacy	169 237 (81.1)	20 282 (9.7)	19 052 (9.1)	208 571^a^ (100.0)	161 346 (81.7)	22 484 (11.4)	13 645 (6.90)	197 475 (100)	<0.001

^a^This was filtered to exclude checklists where multiple answers were ticked, i.e. if ‘yes’ and ‘no’ were selected.

Statements with the lowest patient self-declared knowledge, i.e. where patients ticked ‘no, I do not understand’, were: ‘I know about the side effects that I might get whilst I am on antibiotics’(16.6% in 2021–22 and 18.1% in 2023–24); ‘I know whether my antibiotics should be taken with or without food’ (15.5% in 2021–22 and 16.8% in 2023–24); ‘I know how long my symptoms are likely to last’ (15.5% in 2021–22 and 16.0% in 2023–24); and ‘I know whether I need to avoid alcohol whilst I am taking my antibiotics’ (13% in 2021–22 and 14.6% in 2023–24). Patients presented more self-declared knowledge of AMS-themed statements, for example, ‘I know why I must never share my antibiotics or keep for later use’ (85.9% in 2023–24) and ‘I know why I must return any unused antibiotics to the pharmacy’ (81.7% in 2023–24).

### Patients’ recent antibiotic use

Patients were asked to indicate if they had received the same antibiotic in the previous 3 months (Table 4). If patients ticked ‘yes’, pharmacy staff were advised to have a conversation with these patients about AMR. Across both years, pharmacy staff reported having AMR conversations with more patients than those who responded ‘yes’. In 2021–22, 36 097 (17.5%) of patients reported having the same antibiotic in the last 3 months. In the same year, pharmacy staff reported they had conversations with 45 798 (23%) of patients about AMR after checking that their patient medication records confirmed that they had had the same antibiotic in the previous 3 months. In contrast, in PQS 2023–24, 24 306 (12%) of patients reported having the same antibiotic in the last 3 months, whist pharmacy staff had conversations with 87 975 (44%) of patients about resistance.

**Table 4.** Responses from both patient and pharmacist regarding using the same antibiotic in the last 3 months (n = number of checklists)

	Patient-reported receiving the same antibiotic(s) within previous 3 months, number of checklists, *n* (%)			Pharmacist-reported AMR conversation with patient who received prescription of the same antibiotic(s) within previous 3 months, number of checklists, *n* (%)		
Response	2021–22	2023–24	*P* value	2021–22	2023–24	*P* value
Yes	36 097 (17.5)	24 306 (12)	<0.001	45 798 (23)	87 975 (44.0)	<0.001
No	156 836 (76.0)	159 997 (81)	<0.001	49 153 (25)	35 168 (17.6)	<0.001
Not applicable	0	0	N/A	102 524 (52)	67 210 (33.7)	<0.001
Do not know/missing	13 203 (6.4)	13 172 (7)	<0.001	0	9320 (4.7)	<0.001
Total checklists	206 136	197 475		197 475	199 673^a^	

^a^In PQS 2021–22, this was filtered to exclude checklists where multiple answers were ticked, i.e. if ‘yes’ and ‘no’ were ticked.

### Pharmacy staff interventions on antibiotic prescriptions

Table 5 shows the number and types of interventions made by the pharmacy teams in both schemes. In 2021–22, there were 2741 interventions, where the most frequent reasons reported were ‘unknown/missing’ (1040); strength, dose, duration, quantity, formulation (817) and allergy detected (241). In contrast, there were 1749 interventions in 2023–24; the most frequent reasons were strength, dose, duration, quantity, formulation (724), allergy detected (253) and recent antibiotic use (207). In addition, a further 70 612 infection-specific patient information leaflets (PILs) were given to patients to support discussions around self-care and safety-netting for symptoms during the PQS 2023–24, showing an increase from 69 861 in 2021–22.

**Table 5.** Interventions^a^ made by pharmacy teams

Interventions by pharmacy teams	PQS 2021–22	PQS 2023–24
Allergy detected, *n*	241	253
Medicines interaction, *n*	60	61
Strength, dose, duration, quantity, formulation, *n*	817	724
Multiple antibiotics prescribed, *n*	60	51
Choice of antibiotic for indication, *n*	138	89
Recent antibiotic use, *n*	147	207
Long-term use, prophylaxis, rescue pack, *n*	49	58
Possible unnecessary antibiotic, *n*	22	25
Pregnancy, breast feeding, kidney issue, *n*	73	73
Patient factors (adverse reaction, preference), *n*	26	29
Safety-netting/referral, *n*	18	25
Other, *n*	50	154
Unknown/missing, *n*	1040	196
Total, *n*	2741	1749
Additional infection PIL provided	69 861	70 612

^a^Interventions can be defined as detecting medication errors and/or rationalizing the therapy, which can result in the prescriber being contacted and a change in the prescription if appropriate. Unknown/missing relates to where pharmacists reported they contacted a prescriber about a prescription but did not provide a reason.

There was a small, but statistically significant, increase in almost all safety checks carried out by the pharmacists with the exception of the antibiotic being checked against local guidance, captured using the TARGET antibiotic checklist (Table 6).

**Table 6.** Proportion of antibiotic safety checks completed by the pharmacist or pharmacy team, prompted by the TARGET antibiotic checklist

Intervention	2021–22, *n* (%)	2023–24, *n* (%)	*P* value
Antibiotic dose	200 850 (95)	190 571 (97)	< 0.001
Appropriateness of antibiotic for the infection	200 520 (95)	189 107 (96)	< 0.001
Antibiotic duration	199 989 (94)	190 112 (96)	< 0.001
Patient allergies, risk factors, interactions	198 611 (94)	186 761 (95)	< 0.001
Antibiotics against local guidance	187 754 (89)	173 828 (88)	< 0.001
If the patient had the antibiotic in the last 3 months	171 924 (81)	169 116 (86)	< 0.001

### Vaccination uptake and provision through pharmacy

An overall decline in patient-reported vaccination uptake from 72% (39 960) in 2021–22 to 65.5% (32 770) in 2023–24, and pharmacist-reported on-site vaccination administration from 8% (16 625) in 2021–22 to 6% (11 273) in 2023–24 was observed across the 2 years of the scheme. There was a statistically significant decline in uptake for all categories of patients who would be entitled to a free NHS influenza vaccination, except patients aged 65 years and over (Table 7).

**Table 7.** The number of TARGET antibiotic checklists indicating patients were eligible to receive a free influenza vaccination

Influenza vaccination eligibility category	Number of patients (% of all patients)		Number of influenza vaccines already received within the last 12 months (% by eligibility criterion)		Gave the patient an influenza vaccine on site (% of number of total patients)		*P* value for ‘vaccines already received’ (% by eligibility criterion)	*P* value for ‘gave the patient an influenza vaccine on site’ (% of total number of patients)
	2021–2022	2023–24	2021–2022	2023–24	2021–2022	2023–24		
All patients	208 858 (100.0)	197 475 (100.0)	39 960 (76.0)	32 770 (66.0)	16 625 (8.0)	11 273 (6.0)	<0.001	<0.001
Over 65 years old	47 622 (23.0)	44 833 (22.0)	36 712 (77.0)	30 550 (68.0)	4431 (9.0)	4336 (10.0)	<0.001	0.058
Problem with kidney function^a^	2968 (1.4)	2424 (1.3)	1648 (56.0)	1084 (43.0)	265 (9.0)	180 (7.0)	<0.001	0.051
Problem with liver function^a^	740 (0.6)	577 (0.3)	388 (52.0)	268 (47.0)	85 (11.0)	44 (8.0)	<0.05	<0.05
Pregnant^a^	2262 (1.1)	1830 (0.9)	1212 (54.0)	868 (47.0)	238 (11.0)	155 (8.0)	<0.001	<0.05
Total eligible patients	52 592 (26.0)	49 664 (25.0)	39 960 (76.0)	32 770 (66.0)	5019 (10.0)	4715 (9.0).		

Some patients were eligible for an influenza vaccination by having multiple risk factors, i.e. if a patient had both kidney problems and were over 65 years old. These patients were counted once (as over 65 years old).

^a^Under 65 years old.

## Discussion

A total of 406 333 patients were reviewed using the TARGET antibiotic checklist, with 6209 community pharmacies participating in this AMS domain for both schemes and 10 081 participating in either one of the schemes. In the majority of instances, the patient themselves collected the prescribed antibiotics [78% (163 446) in 2021–22 and 79% (155 697) in 2023–24] in both years, with approximately half of patients taking other medication concurrently. Twelve percent of patients reported an antibiotic allergy in 2021–22, compared with 6% in 2023–24. The most common infection types for both years were respiratory and urinary tract infections, with amoxicillin and nitrofurantoin being the most frequently prescribed antibiotics, respectively. There was a statistically significant increase (23% to 44%) in the proportion of consultations where pharmacy teams had conversations with patients about resistance, initiated as a result of the same antibiotics being prescribed in the last 3 months. In 2021–22, an additional 16 625 influenza vaccinations were delivered by community pharmacies prompted by discussions whilst using the antibiotic checklist (8% of patients audited). In 2023–24, 11 273 additional influenza vaccinations were administered (6% of patients). In 2021–22, in total, 69 861 PILs were provided to patients to increase their knowledge about their condition and treatment. In 2023–24, this had increased slightly to 70 612 PILs. The reasons why pharmacists intervened and queried prescriptions were similar across both years and were for antibiotic dose, appropriateness of antibiotic for the infection, antibiotic duration, patient allergies, risk factors, interactions, antibiotics not being prescribed as per local guidance and the patient having had the antibiotic in the last 3 months,

### Changes in antibiotic prescribing and allergy status

Fewer antibiotic checklists were reviewed for dental infections [15% (30 764) in 2021–22 versus 12.1% (24 440) in 2023–24], whilst more were reviewed for throat infections [11% (22 513) in 2021–22 versus 11.8% (23 784) in 2023/24]. This aligns with national trends in antibiotic prescribing for dental infections following a peak during the COVID-19 pandemic, when dentists were unable to conduct consultations, whilst having to manage patients and their symptoms with the use of antibiotics.^5^ A national increase in phenoxymethylpenicillin prescribing, attributed to increased rates of scarlet fever and invasive group A *Streptococcus* cases, was also observed in this time frame.^14^ The decrease in patients reporting allergies to antibiotics in 2023/24 compared with 2021/22 may be due to an initiative in England to encourage determination of the true allergy status of patients, where evidence has shown that the majority of patients listed to have a penicillin allergy are not truly allergic.^15^

### Patient education and understanding

Outcome 2 of the UK NAP involves public engagement and education with a specific and measurable metric: ‘by 2029, we aim to increase UK public and healthcare professionals’ knowledge on AMR by 10%, using 2018 and 2019 baselines, respectively’.^1^

Whilst our findings demonstrate an increase in the number of patients stating ‘no they did not understand’ for the knowledge statement from 2021–22 to 2023–24, reassuringly ≥75% of patients stated ‘yes, they did know’ to all the statements in both years. A reduction in ‘no response’ to knowledge questions may imply that patients and the public desire more advice about their treatment and symptoms. There has been a switch from ‘no’ responses to ‘no, I do no understand’, where more patients are stating when they don’t understand for all statements rather than giving no response. The statements that patients were least likely to understand across both years were around awareness of side effects, awareness of food instructions for their antibiotics, and awareness of duration of symptoms. These topics are important to highlight when promoting patient education and understanding on antibiotic use.

From 2021–23 to 2023–24, the percentage of patient-reported ‘same antibiotic’ decreased from 17% to 12% but the pharmacist-reported AMR conversations increased from 23% to 44%. This suggests that patient recall of prior antibiotic courses has deteriorated, whilst pharmacist detection of prior antibiotics has improved. The response from the pharmacists shows there is a statistically significant difference in the number of conversations pharmacy teams had with patients about AMR that were initiated as a result of the same antibiotics being prescribed in the last 3 months, which is promising. However, an increase in prior exposure to antibiotics from 23% to 44% is unlikely and it seems more likely that pharmacists were having conversations with patients about AMR irrespective of whether they had had recent prior antibiotics. An amendment to the TARGET antibiotic checklist would be helpful to separate the pharmacist question into two: (i) Do pharmacy records indicate the patient has had the same antibiotic within the last 3 months; and (ii) Did the pharmacy team have a conversation with the patient about AMR? Further prior antibiotic exposure resulted in only 207 interventions.

### Vaccination uptake

Since 1974, vaccinations have been the single greatest contribution to improving mortality globally, particularly for childhood diseases.^16–18^ Vaccination rates are a measure of the strength of a country’s healthcare system, being one of the most significant public health interventions (aside from clean drinking water and sanitation) responsible for better health outcomes globally.^19^ Community pharmacies play a central role in vaccination uptake and delivery. To date, community pharmacies in England are commissioned to provide COVID-19 and influenza vaccinations, with a possible expansion to respiratory syncytial virus (RSV) vaccinations.^20^ Influenza vaccinations are particularly important for older and more vulnerable patients as they are likely to suffer from immunosenescence, i.e. as people age, both adaptive and innate immune systems tend to lose efficacy over time, leading to difficulties in mounting immune responses against new pathogens.^21^ Compared with 2021–22, fewer influenza vaccinations were given by pharmacies to patients onsite in 2023–24 from conversations initiated using the TARGET antibiotic checklist, and this aligns with national influenza vaccination trends. In 2021, England saw the most successful influenza vaccination programme in its history.^22^ Despite the challenges due to the COVID-19 pandemic, at the end of February 2021, NHS services had vaccinated a record 80.9% of those aged 65 years and over in England; the highest uptake ever achieved for this group, exceeding the WHO uptake ambition of 75%.^22^ In 2022 and 2023, the rates had fallen to 79.9% and 77.8% respectively.^23^

Collectively, community pharmacy teams still delivered an extra 27 898 vaccinations cumulatively between both schemes. Research suggests a trust relationship between patients and providers has been shown to reduce influenza vaccine hesitancy.^24^ In the PQS 2023–24, a total of 11 273 extra influenza vaccines were administered, prompted by conversations initiated using the checklist, of which 4805 were to patients who were identified to have higher risk (i.e. over 65 years, pregnant etc.). Considering these findings, the TARGET antibiotic checklist could be updated to ask about all national vaccinations, including COVID-19 and RSV, in order to promote conversations to remind patients to receive these.

### Impact of community pharmacy

There was a statistically significant increase in all clinical review of medication conducted by pharmacists (except checking antibiotics against local guidance), with the majority of these results being in the upper 90th percentile. The number of interventions are small compared with the number of checklists. Further there were fewer interventions in the second year compared with the first year. Possible explanations for this could be improved quality of prescribing or workload pressures. Pharmacists are the third most common profession in the healthcare provider workforce.^25,26^ In England, community pharmacies are often open for extended and unsociable hours, where patients can access services without pre-booking appointments. Since 2021–22, community pharmacy teams have been incentivized to engage in AMS activities via the PQS, which has resulted in education and training to occur at pace for the majority of the community pharmacy workforce.^8,12,27^ The use of the TARGET antibiotics toolkit supports pharmacists to have structured conversations with patients and the public, as well as providing reassurance and safety-netting. The criteria incentivized over the years as part of the PQS are aimed at providing a sound foundation in AMS for community pharmacy teams, giving confidence and reassurance in their ability to manage a wider cohort of patients as part of the Pharmacy First Service launched in January 2024.^8,27–29^ The data from the PQS 2023–24 participation was lower in 2023–24 with less patient data capture compared with 2021–22. Although the scheme is voluntary, the majority of community pharmacy contractors have engaged in the scheme since its inception in 2016, participating in one or more of the domains.^30^ In 2023–24, this number fell to the lowest uptake, with 8625 community pharmacies completing one or more of the domains. The lack of engagement in PQS 2023–24 may be due to challenges in the financial investment to proceed with the scheme and the lack of capacity to take on additional services.^31^

### Strengths and limitations

Over 400 000 patients were reviewed using the TARGET antibiotic checklist, with more than 10 000 community pharmacy teams participating in either year of the PQS. There were minor amendments to the digitalized data collection tool for the 2023–24 PQS, preventing contractors from answering some questions illogically and/or bypassing questions. However, this change compromised a comparison for much of the data to identify any statistically significant difference. It is recognized that community pharmacy contractors are likely to behave differently when they are incentivized but the purpose of the re-incentivization was to embed the use of these tools into everyday practice. The 2023–24 PQS participation was not as the same previous schemes due to the lack of capacity and financial investment.^31^ Further studies could involve extrapolating the use of the TARGET antibiotic checklist with the launch of the Pharmacy First Service (31 January 2024).

## Conclusions and implications

In England, the majority of antibiotics are typically being prescribed in general practice whilst being dispensed in community pharmacy. The PQS investment in training and resource provisions for community pharmacy has showed significant improvements in AMS. Community pharmacy teams were able to further help and educate patients on their knowledge of antibiotic use and their knowledge about symptom resolution and AMS using the TARGET antibiotic checklist, as well as other resources from the TARGET toolkit. The study demonstrates the impact of the TARGET resources, which although developed for the community setting, may be adapted for the wider healthcare system and different sectors, e.g. outpatient pharmacies in hospital. This contribution supports the overall NAP ambition for public engagement and education to achieve an increase in public and healthcare professionals’ knowledge in AMR by 10%. The use of the TARGET antibiotic checklist facilitated by the PQS further demonstrates the key position that community pharmacy teams have in clinically aiding AMS within the community and educating patients about antibiotic use.

## Data Availability

The data presented in this study are available upon reasonable request from the corresponding author.
